# Annexin A2 Promotes the Migration and Invasion of Human Hepatocellular Carcinoma Cells *In Vitro* by Regulating the Shedding of CD147-Harboring Microvesicles from Tumor Cells

**DOI:** 10.1371/journal.pone.0067268

**Published:** 2013-08-12

**Authors:** Wei Zhang, Pu Zhao, Xiu-Li Xu, Lei Cai, Zhen-Shun Song, Da-Yong Cao, Kai-Shan Tao, Wen-Ping Zhou, Zhi-Nan Chen, Ke-Feng Dou

**Affiliations:** 1 Department of Hepatobiliary Surgery, Xijing Hospital, Fourth Military Medical University, Xi'an, Shaanxi Province, China; 2 Department of Hepatobiliary Surgery, General Hospital of Shenyang Military Area Command, Shenyang, Liaoning Province, China; 3 College of Life and Health Sciences, Northeastern University, Shenyang, Liaoning Province, China; 4 Cell Engineering Research Center and Department of Cell Biology, State Key Laboratory of Cancer Biology, State Key Discipline of Cell University, Fourth Military Medical University, Xi'an, Shaanxi Province, China; 5 Center of Clinical Laboratory Medicine of People's Liberation Army, Xijing Hospital, Fourth Military Medical University, Xi'an, Shaanxi Province, China; Seoul National University, Korea, Republic of

## Abstract

It has been reported that Annexin A2 (ANXA2) is up-regulated in hepatocellular carcinoma (HCC), but the roles of ANXA2 in the migration and invasion of HCC cells have not been determined. In this study, we found that ANXA2-specific siRNA (si-ANXA2) significantly inhibited the migration and invasion of HCC cells co-cultured with fibroblasts *in vitro*. In addition, the production of MMP-2 by fibroblasts cultured in supernatant collected from si-ANXA2-transfected HCC cells was notably down-regulated. ANXA2 was also found to be co-localized and co-immunoprecipitated with CD147. Further investigation revealed that the expression of ANXA2 in HCC cells affected the shedding of CD147-harboring membrane microvesicles, acting as a vehicle for CD147 in tumor-stromal interactions and thereby regulating the production of MMP-2 by fibroblasts. Together, these results suggest that ANXA2 enhances the migration and invasion potential of HCC cells *in vitro* by regulating the trafficking of CD147-harboring membrane microvesicles.

## Introduction

The annexins are a family of Ca^2+^-dependent phospholipid-binding proteins with various membrane-related functions. The name “annexin” is derived from the Greek “annex,” which means “bring/hold together,” and was chosen to describe the principal property of all, or at least nearly all, annexins — the binding to and possibly holding together of certain biological structures, in particular membranes [1]. At least 20 members of the family have been described to date [2]. Annexin A2 (ANXA2), also called Annexin II, is one of the best characterized of the Annexins. ANXA2 is composed of two main structural domains: the 33-kDa C-terminal conserved core domain, which contains the Ca^2+^- and membrane-binding sites [1], [3]; and the 3-kDa N-terminal variable domain, which contains the protein binding sites and phosphorylation sites. Otherwise, the N-terminus harbors a high affinity hydrophobic interaction site for the EF-hand Ca^2+^ binding protein S100A10 (p11). Two molecules of ANXA2 and two molecules of p11 form a heterotetrameric complex (A2t) that has been suggested to be involved in exocytosis, endocytosis and membrane vesicle trafficking [4]–[6]. ANXA2 was first discovered as a substrate of the Rous sarcoma virus-encoded tyrosine protein kinase. Subsequent studies have implicated ANXA2 in several biological functions including mitogenic signal transduction [7], fibrinolysis [8], immune response [9], proliferation [10], carcinogenesis and tumor progression [6], [9], [11]–[14]. Large-scale genomic and proteomic studies have begun to accumulate evidence regarding the association and possible involvement of ANXA2 with benign and malignant neoplasms of diverse origins [15]. Increased expression of ANXA2 has been described in a large number of spontaneous neoplasms, including pancreatic cancer, gastric carcinoma, colorectal cancer, breast cancer, high-grade gliomas and kidney cancer (reviewed in [3]) and is positively correlated with tumor invasion and migration [16]. In contrast, the expression of ANXA2 is lost or reduced in prostate cancer, and the role of ANXA2 in prostate cancer appears contradictory [17], [18]. The differential expression of ANXA2 in HCC and normal liver tissue has been reported, but a more detailed functional assessment is lacking [19].

Although published data support a crucial role for ANXA2 in tumor progression, the detailed mechanisms underlying this role have yet to be fully elucidated. Breakdown of the extracellular matrix (ECM), which is mediated by a variety of proteases, endows malignant cells with the ability to penetrate through tissue barriers and is believed to play a major role in tumor migration and invasion. ANXA2 has been found to be a putative co-receptor for both plasminogen and tissue-type plasminogen activator (tPA) [20]. Cell surface ANXA2 acts as a platform for plasmin activation, where inactive plasminogen is cleaved by tPA to yield the active serine proteinase, plasmin, thereby facilitating the migration and invasion of malignancies. Studies have also demonstrated that ANXA2 may regulate the production and activation of matrix metalloproteinases (MMPs) [20], [21].

CD147 is a widely distributed cell surface glycoprotein that belongs to the immunoglobulin superfamily. It was first identified as a factor shedding from the surface of tumor cells that is responsible for stimulating the production of MMP-1 by fibroblasts [22]. Accumulating evidence indicates that CD147 is a major mediator of the malignant phenotypes of various tumors [23]. CD147 induces angiogenesis by stimulating the production of VEGF, invasiveness by stimulating the production of MMPs and multidrug resistance via hyaluronan-mediated up-regulation of ErbB2 signaling and the activity of cell survival pathways [24]. Induction of MMP production through cell interactions is one of the most important functions of CD147 — thus the derivation of its other name: **e**xtracellular **m**atrix **m**etallo**pr**oteinase **in**ducer (EMMPRIN) [25]. CD147 may serve as its own counter-receptor in homotypic cancer cell interactions and cancer cell-fibroblast interactions, thereby stimulating the production of MMPs via a homophilic interaction with other CD147 proteins [26], [27]. In addition, MT1-MMP, MMP-2, and MMP-9 have been reported to cleave and release a shorter form of soluble CD147 that lacks the C-terminus, thereby modulating the expression of MMPs [26], [28]. Interestingly, recent studies have provided evidence that membrane microvesicles shed from tumor cells carry full-length CD147 and play a role in tumor–stromal interactions through the upregulation of the production of MMPs [29], [30]. Previous studies have demonstrated that CD147 promotes the invasion and metastasis of human hepatoma cells by stimulating both tumor cells and peritumoral fibroblasts to produce elevated levels of MMPs, although the modulation of fibroblasts is the more critical part of the process [31], [32].

Although the overexpression of ANXA2 in HCC has been shown, the role of ANXA2 in the migration and invasion of HCC cells remains obscure [33], [34]. In the present study, we knocked down the expression of ANXA2 in HCC cells to explore its role in HCC cell migration and invasion. To further investigate the mechanisms of ANXA2 in tumor progression, we introduced CD147, which has been hypothesized to interact with ANXA2 but which has not yet been shown to do so [24]. Based on the involvement of ANXA2 in exocytosis and membrane vesicle trafficking [4], [5] and the role of CD147-harboring membrane microvesicles on tumor progression, we hypothesized that ANXA2 is involved in the shedding of CD147-harboring microvesicles from tumor cells, thereby regulating tumor migration and invasion.

## Materials and Methods

### Cell lines

Two highly invasive human HCC cell lines, SMMC-7721 and FHCC-98, were cultured in DMEM containing 10% fetal bovine serum (FBS). SMMC-7721 cells were obtained from the Shanghai Institute of Biochemistry and Cell Biology, Chinese Academy of Science [32]. The FHCC-98 cells were purchased from the Cell Engineering Research Centre, Fourth Military Medical University, China [34]. Human embryo pulmonary fibroblast-1 (HPF-1) cells were purchased from the Chinese Academy of Medical Sciences and were cultured as described above [31].

### RNA interference

si-ANXA2 was purchased from Santa Cruz Biotechnology, Inc. (sc-29199) [33]. si-CD147 (5′-GUUCUUCGUGAGUUCCUCtt-3′, 3′-dTdTCAAGAAGCACUCAAGGAG-5′) was synthesized by Ambion, Inc [31]. HCC cells were transfected with siRNA using LipofectAMINE 2000 according to the manufacturer's instructions (Invitrogen, USA). Silencer negative control siRNA (snc-RNA) (Ambion, USA) was used as a negative control under identical conditions.

### Reverse transcription-PCR

Forty-eight hours after transfection with siRNA, total RNA was extracted from the cells with TRIzol (Invitrogen, USA) and reverse transcribed into cDNA using a ReverTra Ace-α-™ kit (TOYOBO, Japan). GAPDH was used as an internal control. All primers were synthesized by Shanghai Sangon Co. as follows: ANXA2, forward primer 5′-GAGGATGGCTCTGTCATTGATT-3′; reverse primer 5′-CTGGTAGTCGCCCTTAGTGTCT-3′; GAPDH, 5′-ACCACAGTCCATGCCATCAC-3′ and 5′-TCCACCACCCTGTTGCTGTA-3′.The conditions for PCR were one cycle at 94°C for 4 min, 40 cycles at 94°C for 30 s, 60°C for 30 s, and 72°C for 30 s. PCR products were electrophoresed on 1% agarose gels. All PCR reactions were performed in triplicate.

### Western blot

Cells were harvested and lysed in lysis buffer. A BCA Protein Assay Kit (Pierce Biotechnology, USA) was employed to determine the concentration of total protein. Equal amounts of protein were separated by SDS-PAGE (12%). Proteins were transferred to a polyvinylidene fluoride (PVDF) microporous membrane (Millipore, USA) and the blots probed with ANXA2 mAb (Santa Cruz, USA) or CD147 mAb (Santa Cruz, USA). Tubulin was chosen as an internal control, and the blots were probed with mouse anti-tubulin mAb (Santa Cruz, USA).

### *In vitro* migration/invasion assays

The assays were performed using chambers with polycarbonate filters (pore size, 8 µm). Some filters were coated with Matrigel (Becton Dickinson Labware, USA), and some were not. Twenty-four hours after being transfected with siRNA, HCC cells were harvested. Equal numbers (5×10^4^) of transfected and HPF-1 cells were then placed in the upper chamber in 300 µL of 0.1% serum medium. Cells transfected with snc-RNA were used as a negative control. The lower chamber was filled with 0.1% fetal bovine serum medium (200 µL) and serum-free conditioned medium collected from HPF-1 cells (200 µL). After 8 h (migration assay) or 24 h (invasion assay) of incubation, the cells in the upper chamber were carefully removed with a cotton swab. The cells on the underside were then fixed in methanol, stained with H&E, and counted under a microscope.

### Gelatin Zymography

Serum-free conditioned medium collected from siRNA-transfected SMMC-7721 and FHCC-98 cells was added to HPF-1 cells. Fifteen hours later, the conditioned medium was collected and separated using a 10% acrylamide gel containing 0.1% gelatin. The gels were incubated in a 2.5% Triton X-100 solution at room temperature with gentle agitation and were then soaked in reaction buffer [0.05 mol/L Tris-HCl (pH 7.5), 0.2 mol/L NaCl, and 0.01 mol/L CaCl_2_] at 37°C for 18 h. After the reaction, the gels were stained for 6 h with Coomassie brilliant blue and de-stained for 0.5 h. Areas of gelatinolytic activity were evident based on their negative staining.

### Immunocytochemistry and confocal microscopy

SMMC-7721 and FHCC-98 cells were harvested and allowed to attach to pre-coated glass coverslips for 24 hours. They were then fixed in 3.7% formaldehyde in PBS, permeabilized with 0.1% Triton X-100 and blocked with 1% BSA (Fraction V) in PBS for 1 h. Coverslips were incubated with goat anti-CD147 antibody (1∶100; Santa Cruz, USA) and ANXA2 mAb (1∶100) in PBS overnight at 4°C. The cells were then washed and incubated with Alexa 594-conjugated goat anti-mouse (Invitrogen, USA) or donkey anti-goat IgG-FITC (Santa Cruz, USA) secondary antibody at a dilution of 1∶400 for 1 h at room temperature. Cell nuclei were stained with DAPI (Vector, USA). The slides were then washed and mounted onto glass slides. Anti-fade was added to prevent quenching of the fluorophores. The proteins were visualized with a FluoView™ FV1000 confocal microscope (Olympus, Japan).

### Extraction and co-immunoprecipitation of total cellular membrane proteins

Total cellular membrane proteins (TMP) were extracted from SMMC-7721 and FHCC-98 cells using a Plasma Membrane Protein Extraction Kit (BioVision, USA), according to the manufacturer's instructions. SMMC-7721 and FHCC-98 cells (5×10^7^) were collected, centrifuged and washed with 1 ml ice-cold PBS. The cells were then re-suspended in 1 ml Homogenize Buffer Mix in an ice-cold Dounce homogenizer 30–50 times. The homogenate was centrifuged at 700 g for 10 minutes at 4°C. The supernatant was collected and centrifuged at 10,000 g for 30 minutes at 4°C. The cytosol fraction (supernatant) and total cellular membrane proteins (pellet) were collected. The samples were either used immediately or stored at −20°C for further study.

The interaction of ANXA2 with CD147 in the plasma membranes of SMMC-7721 and FHCC-98 cells was detected using a ProFound™ Mammalian Co-Immunoprecipitation Kit (Pierce, USA) according to the manufacturer's instructions. Briefly, TMP extracted as described above were collected onto a Coupling gel pre-bound with 200 µg anti-ANXA2 mAb, anti-human CD147 mAb, or anti-JEV mAb (mouse IgG, kindly provided by the Department of Microorganism, Fourth Military Medical University and used as a negative control), followed by four washes with the co-immunoprecipitation buffer. The coupling gel was washed with elution buffer, and aliquots of the eluent were used for Western blotting with anti-ANXA2 mAb and anti-CD147 mAb.

### Isolation and electron microscopy of membrane microvesicles

Microvesicles were isolated as previously described [30]. Briefly, medium conditioned for 24 hours by subconfluent, healthy SMMC-7721 cells was centrifuged at 600 g for 15 minutes and then at 1500 g for 15 minutes to remove cells and large debris. The supernatant was ultracentrifuged at 100,000 g for 1 hour at 4°C. Pelleted microvesicles were re-suspended in PBS (pH 7.4). Isolated vesicles were quantified based on protein concentration measurements using the Bradford method (Bio-Rad, Milan, Italy), with BSA (Sigma, St. Louis, MO) used as a standard. Samples were either frozen at −20°C or used immediately for electron microscopy studies. Aliquots of vesicles were applied to 200 mesh nickel parlodion-coated grids and allowed to settle. Samples were negatively stained with 1% phosphotungstic acid (pH 6) before being analyzed with an electron microscope [29].

## Results

### Specific siRNA effectively down-regulates ANXA2 expression in HCC cells

**Figure 1:**
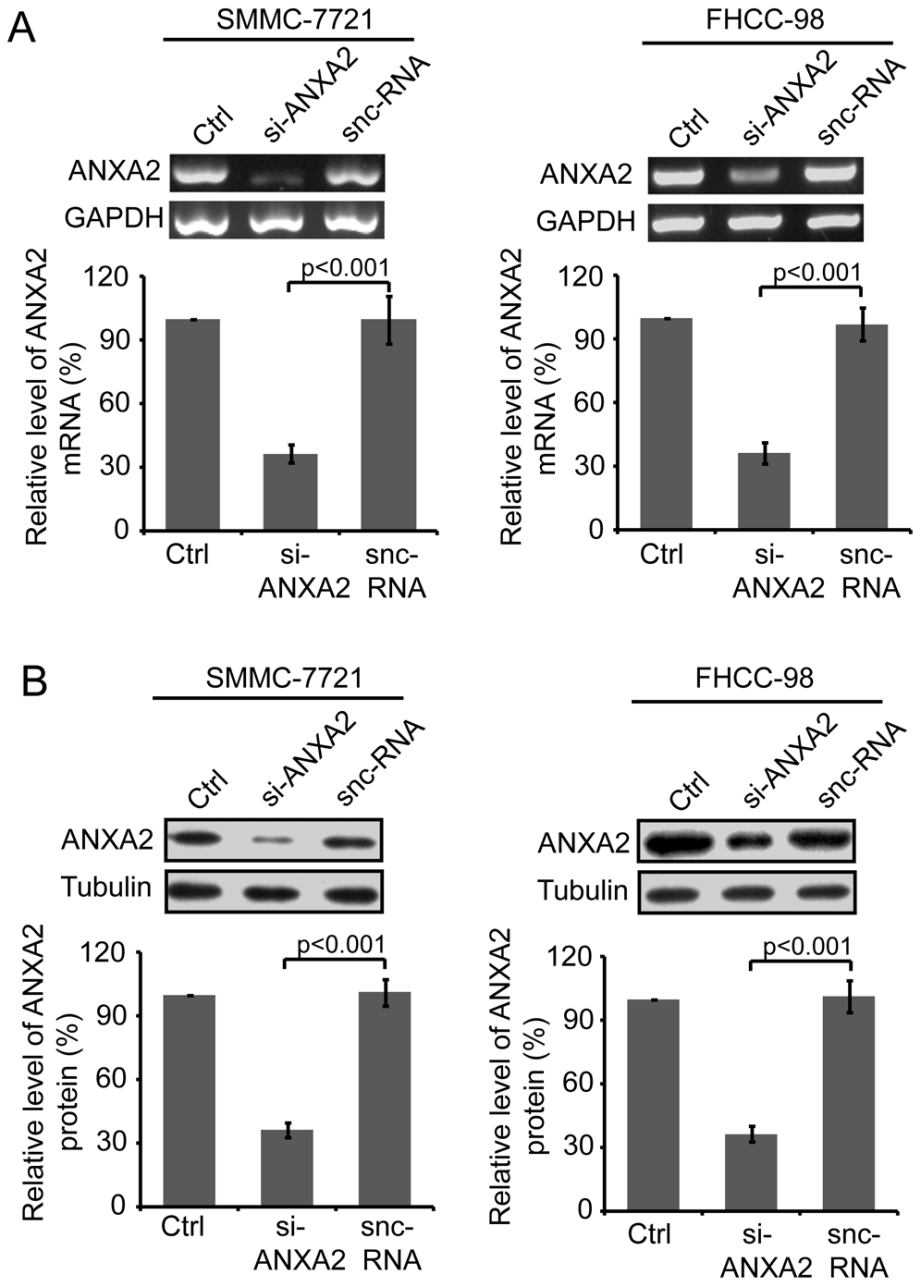
Expression of ANXA2 in HCC cells was effectively down-regulated by specific si-ANXA2. Forty-eight hours after the transfection of SMMC-7721 or FHCC-98 cells with si-ANXA2 or snc-RNA, ANXA2 expression levels were examined using RT-PCR (A) and Western blot (B). Top, representative image; bottom, quantitative gray scale analysis of at least three independent experiments. Columns, mean; bars, SD; *p<0.001* vs. corresponding snc-RNA-transfected cells (one-way ANOVA).

To investigate the role of ANXA2 in the invasion and migration of HCC cells, RNA interference was used to downregulate the expression of ANXA2 in SMMC-7721 and FHCC-98 cells. Both cell lines were transfected with si-ANXA2 and snc-RNA. Forty-eight hours after transfection, the expression of ANXA2 mRNA and protein was examined using RT-PCR and Western blot, respectively. RT-PCR showed that si-ANXA2 could effectively downregulate the expression of ANXA2 mRNA in SMMC-7721 and FHCC-98 cells, with an inhibition rate of 63.39±3.87% and 62.80±2.98%, respectively, compared to snc-RNA (*p<0.001*, Fig. 1A). These results were consistent with Western blot assays. The protein expression of ANXA2 was notably reduced in si-ANXA2 transfected cells, with an inhibition rate of 64.12±1.46% and 58.80±1.36%, respectively, compared to snc-RNA-transfected cells (*p<0.001*, Fig. 1B). These data show that ANXA2-specific siRNA can effectively reduce the expression of ANXA2 in HCC cells.

### Effects of ANXA2 on the migration and invasion of HCC cells

**Figure 2:**
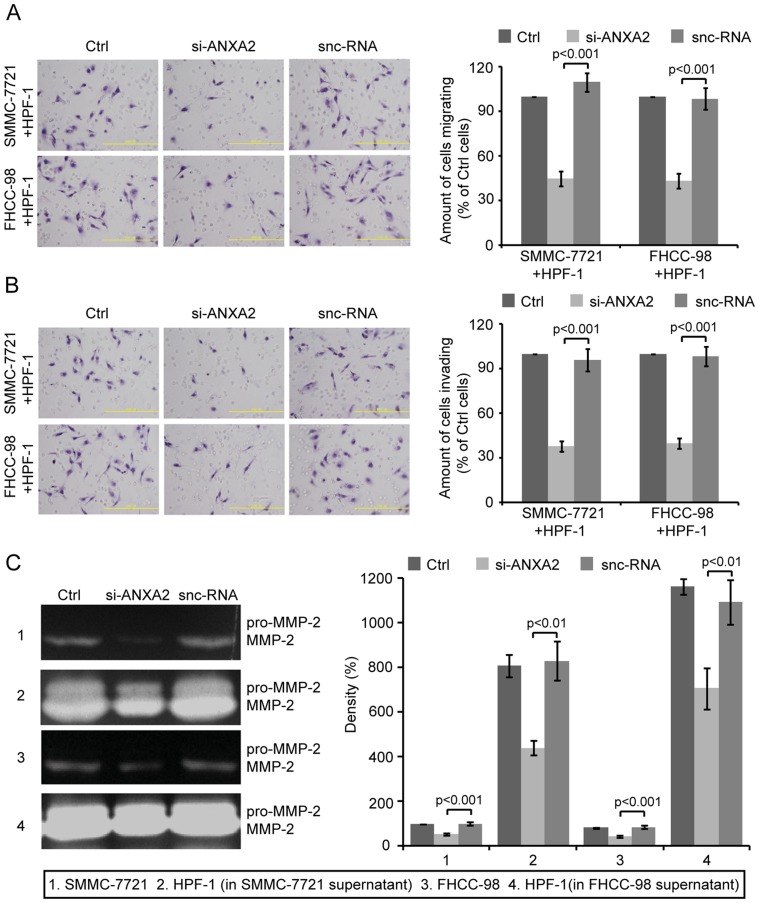
Effects of ANXA2 on the migration and invasion of HCC cells co-cultured with HPF-1 cells. Twenty-four hours after transfection with si-ANXA2 or snc-RNA, HCC cells were co-cultured with an equal number of HPF-1 cells in the upper chamber, which were not coated with Matrigel in the *in vitro* migration assay (A) but were coated in the *in vitro* invasion assay (B). Eight (migration assay) or twenty-four (invasion assay) hours later, the cells migrating/invading through the filter were stained and counted. Left, representative images showing the density of cells on the filter. Right, quantitative analyses of the cells migrating/invading through the filter in three independent experiments. Columns, mean; bars, SD. *p<0.001* vs. corresponding snc-RNA-transfected cells (one-way ANOVA). (C) Silencing ANXA2 in HCC cells inhibited the cellular secretion of MMP-2 by HPF-1 cells cultured in supernatant collected from si-ANXA2-transfected HCC cells. Serum-free conditioned medium collected from si-ANXA2- or snc-RNA-transfected HCC cells was added to HPF-1 cells. Fifteen hours later, the conditioned medium was collected and analyzed using gelatin zymography. Left, representative image. Right, gray scale analysis of at least three independent experiments. Columns, mean; bars, SD. *p<0.01* vs. corresponding snc-RNA-transfected cells (one-way ANOVA).

To mimic *in vivo* tumor-stroma interactions within local microenvironments, HCC cells were transfected with si-ANXA2 and then co-cultured with an equal number of HPF-1 cells in the upper chamber. The migration assay showed that the number of cells that migrated through the filter was drastically reduced in the SMMC-7721 and FHCC-98 cells transfected with si-ANXA2, with an inhibition rate of 59.22±2.43% and 55.94±2.76%, respectively, compared to snc-RNA-transfected cells (*p<0.001*, Fig. 2A). Using an *in vitro* invasion assay, the number of invading cells was also shown to be reduced in si-ANXA2-transfected SMMC-7721 and FHCC-98 cells, with an inhibition rate of 60.63±1.65% and 59.49±3.19%, respectively, compared to snc-RNA-transfected cells (*p<0.001*, Fig. 2B).

Both *in vitro* and *in vivo*, increased tumor aggression has been reported to be correlated with high expression levels of MMPs, which are mainly secreted by stromal cells. A critical question that arises from our study is whether the expression level of ANXA2 in HCC cells affects the production of MMPs by peritumoral fibroblasts. To address this issue, we performed a gelatin zymography assay, which indicated that the secretion of MMP-2 was significantly reduced in HPF-1 cells cultured in supernatant collected from si-ANXA2-transfected HCC cells, with an inhibition rate of 46.75±3.29% and 35.60±2.88% in SMMC-7721 cells and FHCC-98 cells, respectively, compared to snc-RNA-transfected cells (*p<0.01*, Fig. 2C). These data demonstrate that ANXA2 may facilitate the migration and invasion of HCC cells *in vitro* by regulating the production of MMP by peritumoral fibroblasts.

### ANXA2 and CD147 co-localize on HCC membrane structures

**Figure 3:**
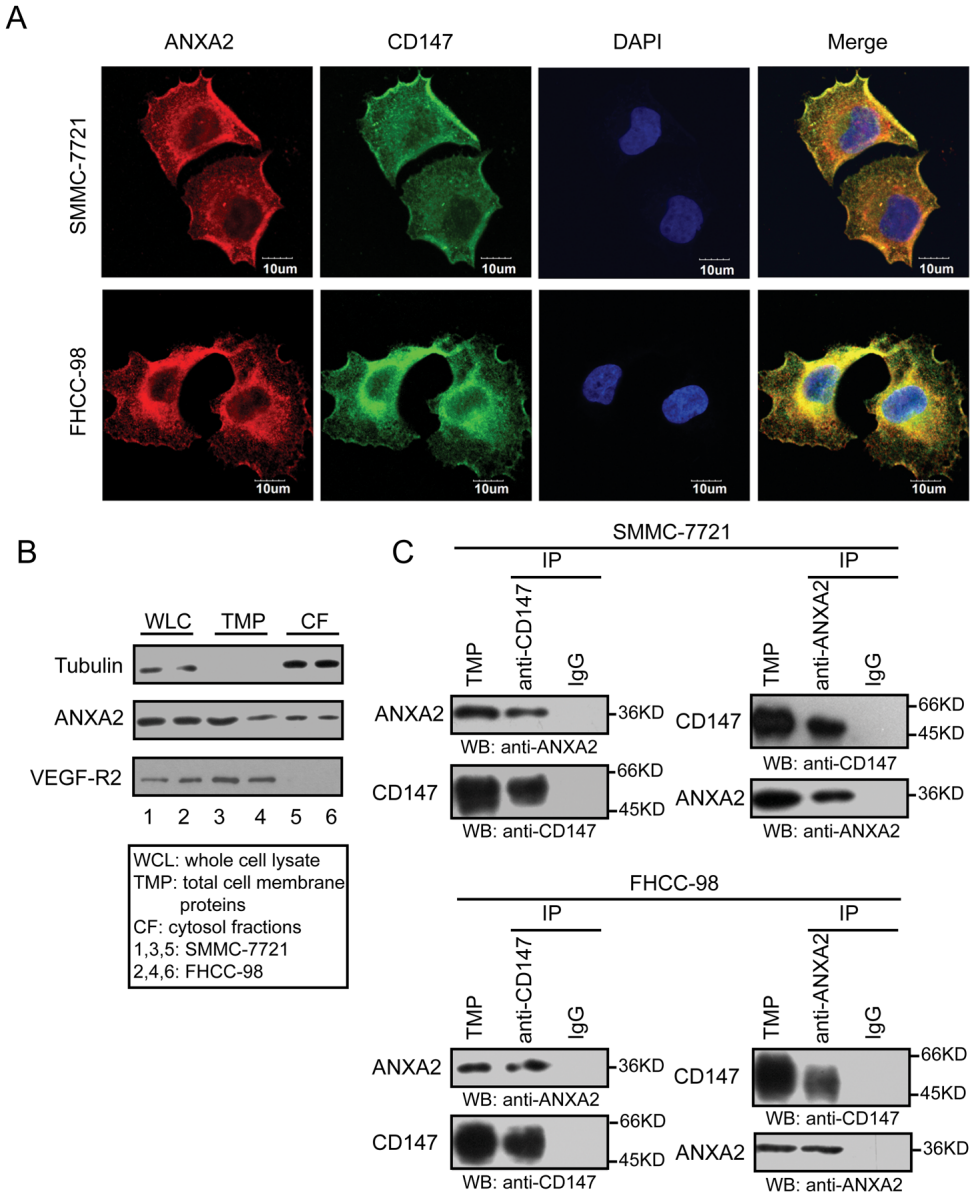
Co-localization and co-immunoprecipitation of ANXA2 and CD147 in SMMC-7721 and FHCC-98 cells. (A) Expression and localization of ANXA2 and CD147 in HCC cells. SMMC-7721 and FHCC-98 cells were double-stained for ANXA2 (red) and CD147 (green). Bar, 10 µm. (B) Total cell membrane protein (TMP) extracted from HCC cells. TMP was extracted from SMMC-7721 cells and FHCC-98 cells using a Plasma Membrane Protein Extraction Kit. The expression of tubulin, VEGF-R2 and ANXA2 in whole cell lysates (WCL), TMP and cytosol fractions (CF) were detected using Western blot. (C) Co-immunoprecipitation of ANXA2 with CD147 in TMP extracted from HCC cells. TMP extracted from SMMC-7721 and FHCC-98 cells was subjected to immunoprecipitation with a coupling gel that was pre-bound with anti-ANXA2 or anti-CD147 antibody. ANXA2 and CD147 in the immune complexes were detected using Western blot. Mouse IgG was used as a negative control.

To study the possible interaction between ANXA2 and CD147 and to explore the hypothesis that ANXA2 is involved in the shedding of CD147-harboring microvesicles from tumor cells, we performed immunofluorescent double-labeling on SMMC-7721 and FHCC-98 cell slides. As shown in Fig. 3A, ANXA2 (red) and CD147 (green) were co-localized in both cell lines. We focused on the strong double-staining of the membrane. To further confirm the results and explore the possibility that ANXA2 is involved in CD147 membrane microvesicle trafficking, we extracted the TMP from SMMC-7721 and FHCC-98 cells. Cytoskeletal protein (tubulin), membrane protein (VEGF-R2), and ANXA2 were detected in whole cell lysates and TMP and cytosol fractions using Western blot (Fig. 3B). We then performed co-immunoprecipitation of the TMP extracted from HCC cells. These results showed that ANXA2 and CD147 co-immunoprecipitated with each other in the TMP extracted from both SMMC-7721 and FHCC-98 cells (Fig. 3C), indicating that ANXA2 and CD147 co-localize on HCC membrane, and there may be interaction effects due to membrane-associated events between the two molecules.

### ANXA2 affects the invasiveness of tumor cells by regulating the transportation of CD147-harboring membrane microvesicles

**Figure 4:**
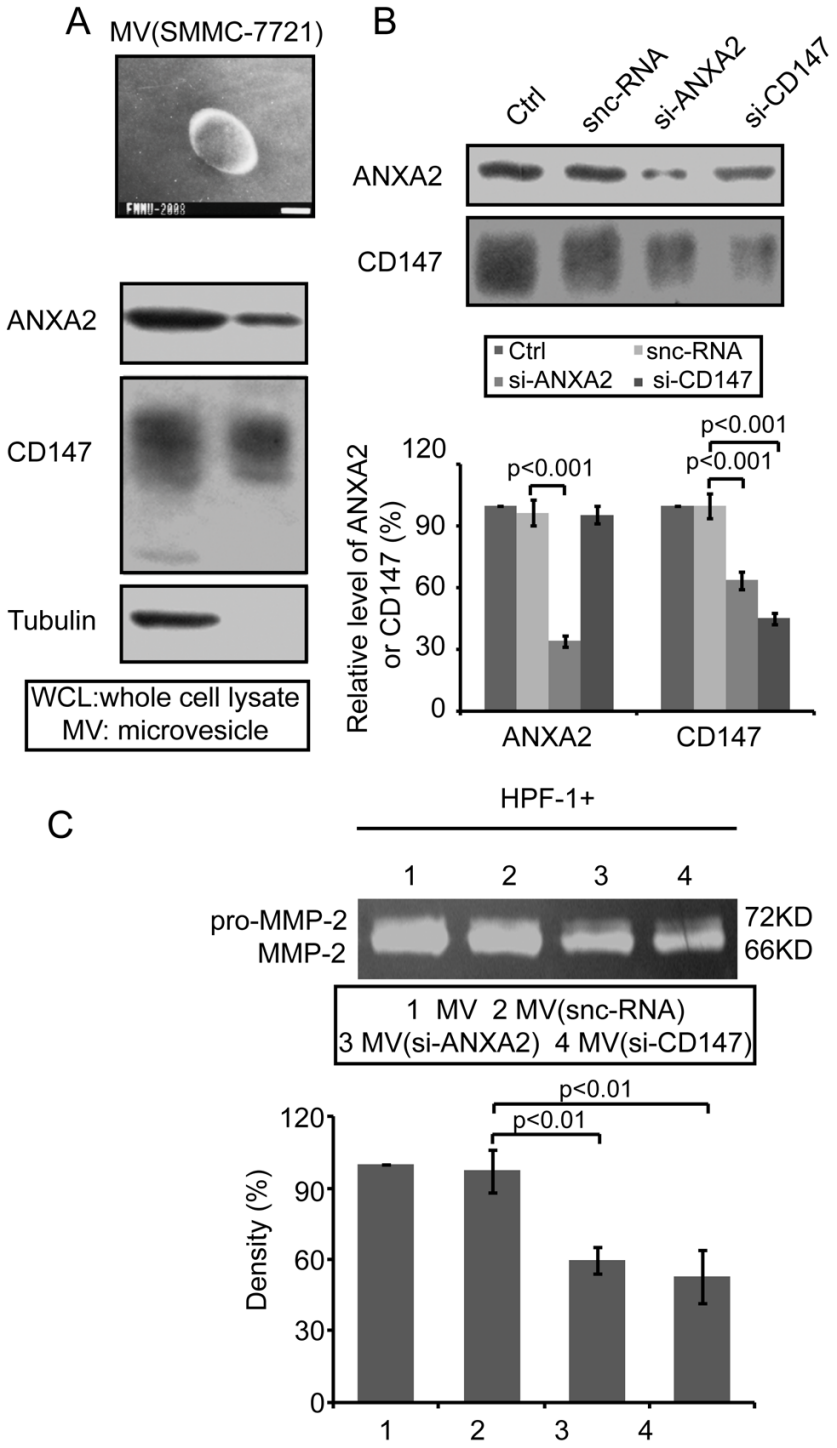
ANXA2 promoted the invasiveness of HCC cells *in vitro* by regulating the transportation of CD147-harboring membrane microvesicles. Microvesicles (MV) were isolated from the supernatant of SMMC-7721 cells using ultracentrifugation and subjected to microscopy (A. Top, representative image of isolated microvesicles. Bar, 100 nm) and Western blot (A. Bottom, representative image). (B) Effects of si-ANXA2 and si-CD147 on the expression of ANXA2 and CD147 in the isolated microvesicles. SMMC-7721 cells were transfected with snc-RNA, si-ANXA2, or si-CD147 and microvesicles then isolated from the supernatant and analyzed using Western blot. Top, representative image of Western blot. Bottom, gray scale analysis of at least three independent experiments. Columns, mean; bars, SD. *p<0.001* vs. corresponding snc-RNA-transfected cells (one-way ANOVA). (C) Effects of tumor-shed CD147-harboring microvesicles on the production of MMP-2 by fibroblasts. SMMC-7721 cells were transfected with snc-RNA, si-ANXA2, or si-CD147 and microvesicles isolated using ultracentrifugation. HPF-1 cells were treated with diverse isolated microvesicles and the conditioned medium then collected and analyzed using gelatin zymography. Top, representative image. Bottom, gray scale analysis of at least three independent experiments. Columns, mean; bars, SD. *p<0.01* vs. corresponding snc-RNA-transfected cells (one-way ANOVA).

Data have shown that ANXA2 is involved in microvesicle trafficking [4] and that CD147-harboring microvesicles play an important role in tumor-stroma cross-talk by stimulating the production of MMPs [29], [30]. The co-localization of ANXA2 and CD147 described above indicated that ANXA2 may be involved in CD147 membrane microvesicle trafficking, and could therefore affect the invasiveness of tumor cells. To address this issue, we isolated microvesicles shed from SMMC-7721 cells and analyzed the harvested pellet using Western blot. Electron microscopy of the ultracentrifuged pellet revealed that SMMC-7721 cells shed spherical or ellipsoid-shaped microvesicles (∼200–500 nm), consistent with previous reports on plasma membrane-derived microvesicles [29]. A representative image is shown in Fig. 4A. The microvesicles were then analyzed using Western blot, which showed that the microvesicles shed from SMMC-7721 cells carried both ANXA2 and CD147 (Fig. 4A).

To further investigate the effect of ANXA2 on the shedding of CD147-harboring microvesicles, SMMC-7721 cells were transfected with si-ANXA2 or si-CD147 and the microvesicles then isolated and analyzed as described above. The results (Fig. 4B) showed that the expression of both ANXA2 and CD147 in the isolated microvesicles was downregulated when SMMC-7721 cells were transfected with si-ANXA2, with an inhibition rate of 64.49±3.56% and 36.16±1.49%, respectively, compared to the snc-RNA-transfected group (*p<0.01*). However, when SMMC-7721 cells were transfected with si-CD147, only the expression of CD147 in the microvesicles was significantly downregulated, with an inhibition rate of 54.78±0.64% compared to the snc-RNA-transfected group (*p<0.01*).

We then focused on the effect of the isolated microvesicles on the secretion of MMPs by fibroblasts. HPF-1 cells were treated with microvesicles isolated from untreated or si-RNA-transfected (snc-RNA, si-ANXA2 or si-CD147) SMMC-7721 cells. A gelatin zymography assay (Fig. 4C) indicated that microvesicles isolated from si-ANXA2- or si-CD147-transfected SMMC-7721 cells were less efficient in the induction of MMP-2 compared to microvesicles isolated from untreated or snc-RNA-transfected SMMC-7721 cells (*p<0.01*). These results suggest that ANXA2 is involved in the trafficking of CD147-harboring microvesicles derived from tumor cells, which regulates the production of MMP-2 by fibroblasts, thereby facilitating the progression of HCC.

## Discussion

ANXA2 is thought to be involved in the transduction of cellular signals associated with inflammation, differentiation and proliferation [2]. Emerging evidence indicates that changes in the expression and/or subcellular localization of ANXA2 contribute to the development and progression of a variety of malignancies. Frohlich et al. reported that ANXA2 was up-regulated in HCC [35]. Recently, Mohammad et al. confirmed the upregulation of ANXA2 in HCC and provided a more detailed description [19]. They reported that ANXA2 is almost undetectable in normal liver and chronic hepatitis tissue, but the expression of ANXA2 is abundant in non-tumorous cirrhotic tissue at both the transcriptional and translational levels. Furthermore, the expression of ANXA2 is greater in tumorous tissue than in non-tumorous cirrhotic tissue. These data strongly indicate that ANXA2 may be involved in the malignant transformation and progression of hepatocellular carcinoma.

In the present study, we explored the role of ANXA2 in the invasion and migration of HCC cells. Two HCC cell lines, SMMC-7721 and FHCC-98, were co-cultured with HPF-1 cells to mimic the tumor-stroma interaction system *in vitro*. A migration assay, invasion assay, and gelatin zymography assay were employed. Our results show that knocking down the expression of ANXA2 in HCC cells inhibits the migratory and invasive potential of these tumor cells and significantly attenuates the production of MMPs by fibroblasts, which were previously reported to be involved in human hepatic tumorigenesis and metastasis. All these findings suggest that ANXA2 may play an important role in the progression of HCC, including migration, invasion, and enzyme degradation.

Previous studies have illustrated that ANXA2 functions in the invasion and migration of various tumors. However, the exact molecular mechanisms underlying this function remain largely unknown. Sharma and Sharma proposed a mechanistic cascade for the role of AXNA2 in tumor progression. They suggested that ANXA2 at the cell surface of tumor/endothelial cells provides for the mechanical assembly of plasminogen activator and plasminogen, which locally activates plasminogen to plasmin. Plasmin then induces the degradation of ECM, which in turn facilitates endothelial/tumor cell invasion and migration [6]. In this study, we found that the treatment of HCC cells with ANXA2-specific siRNA significantly reduced the production of MMP-2 by HPF-1 cells cultured in supernatant collected from HCC cells, suggesting that certain factors may exist in the supernatant that regulate the production of MMPs by HPF-1 cells. Accordingly, we focused on CD147, an important molecule responsible for stimulating the production of several MMPs (MMP-1, MMP-2, MMP-3, MMP-9, MMP-14, and MMP-15) by fibroblasts and endothelial cells [24]. Thus, a possible interaction between ANXA2 and CD147 was considered. In this study, CD147 was found to co-localize with ANXA2 in HCC cells. The two molecules were also found to co-immunoprecipitate with each other in TMP extracted from SMMC-7721 and FHCC-98 cells. These results demonstrate that ANXA2 and CD147 are in close proximity, if not directly associated, and most likely interact in HCC cells.

If we assume that CD147 is the bioactive factor hiding in the supernatant, how and in what form is CD147 released by tumor cells? Vesicle shedding has been observed in normal cells under certain physiological conditions [36] and is present at much higher rates in tumor cells [37]. The shedding of tumor surface antigens in membrane vesicles has been implicated as an important feature of malignant transformation. It is likely that vesicle shedding and, more importantly, the factors released by vesicle shedding, are vital to tumor survival and growth because it is by the release of such factors that tumors condition their microenvironment, regulate metastasis and evade immune surveillance [38]. Sidhu provided evidence of a form of tumor-stromal interaction. He showed that the degradation of the ECM by fibroblasts is controlled by the microvesicular release of CD147 from NCI-H460 cells [29]. In addition, ANXA2 has been shown to participate in the aggregation and transportation of membrane microvesicles [1]. Our present data support the possibility of an interaction between ANXA2 and CD147. We then hypothesized that CD147 carried by membrane microvesicles may be the soluble bioactive factor affecting the production of MMPs in the supernatant collected from HCC cells and that ANXA2 may be involved in the trafficking. Our subsequent study confirmed this hypothesis. Membrane microvesicles were successfully isolated from the supernatant of SMMC-7721 cells using ultracentrifugation. Both CD147 and ANXA2 were detected in the isolated microvesicles using Western blot. When HPF-1 cells were treated with microvesicles, the production of MMP-2 was significantly increased. To further investigate the effect of ANXA2 on the shedding of CD147-harboring microvesicles, SMMC-7721 cells were transfected with si-ANXA2 or si-CD147 and microvesicles then isolated. These results indicate that the downregulation of ANXA2 in tumor cells reduces the expression of CD147 in isolated microvesicles, but the expression of ANXA2 in the microvesicles is not affected when cells are transfected with si-CD147. It has been reported that ANXA2 is involved in exocytosis and membrane vesicle trafficking [4], [5], which may be the reason why the down-regulation of ANXA2 affects CD147 protein levels. There is no evidence that CD147 regulates the production and release of microvesicles, which is consistent with our result that the down-regulation of CD147 did not affect ANXA2 protein levels. The induction effect of microvesicles on the production of MMP-2 was weakened when SMMC-7721 cells were transfected with either si-ANXA2 or si-CD147. All of these findings show that the shedding of microvesicles from tumor cells acts as an efficient vehicle for CD147 trafficking and that ANXA2 regulates the transportation of CD147-harboring microvesicle, thereby contributing to the progression of HCC, although there may be other molecules in the microvesicles besides CD147.

In conclusion, we report that ANXA2 promotes the migration and invasion of HCC cells co-cultured with fibroblasts *in vitro* by regulating the shedding of CD147-harboring microvesicles from tumor cells, which contributes to tumor-stroma crosstalk and sheds light on the mechanisms of ANXA2 in tumor progression. ANXA2 may be a potential target for the development of effective therapeutic strategies for the treatment of HCC.
